# Integrated electronic controller for dynamic self-configuration of photonic circuits

**DOI:** 10.1038/s41377-025-01977-w

**Published:** 2025-09-30

**Authors:** Emanuele Sacchi, Francesco Zanetto, Andres Ivan Martinez, SeyedMohammad SeyedinNavadeh, Francesco Morichetti, Andrea Melloni, Marco Sampietro, Giorgio Ferrari

**Affiliations:** 1 Department of Electronics, Information and Bioengineering, Politecnico di Milano, piazza Leonardo da Vinci 32, Milano, 20133 Italy; 2 Department of Physics, Politecnico di Milano, piazza Leonardo da Vinci 32, Milano, 20133 Italy

**Keywords:** Adaptive optics, Atmospheric optics, Fibre optics and optical communications, Integrated optics

## Abstract

Reconfigurable photonic integrated circuits (PICs) can implement arbitrary operations and signal processing functionalities directly in the optical domain. Run-time configuration of these circuits requires an electronic control layer to adjust the working point of their building elements and compensate for thermal drifts or degradations of the input signal. As the advancement of photonic foundries enables the fabrication of chips of increasing complexity, developing scalable electronic controllers becomes crucial for the operation of complex PICs. In this paper, we present an electronic application-specific integrated circuit (ASIC) designed for reconfiguration of PICs featuring numerous tunable elements. Each channel of the ASIC controller independently addresses one optical component of the PIC, and multiple parallel local feedback loops are operated to achieve full control. The proposed design is validated through real-time reconfiguration of a 16-channel silicon photonics adaptive universal beam coupler. Results demonstrate automatic coupling of an arbitrary input beam to a single-mode waveguide, dynamic compensation of beam wavefront distortions and successful transmission of a 50 Gbit/s signal through an optical free-space link. The low power consumption and compactness of the electronic chip provide a scalable paradigm that can be seamlessly extended to larger photonic architectures.

## Introduction

There has been growing interest and need for general-purpose photonic integrated circuits (PICs) that can be configured to perform all-optical signal processing of light beams^1^. Because of their flexibility, several applications have been proposed for these devices, including reconfigurable filters^2^, microwave photonics and beam forming networks^3^, separation of mixed guided modes^4^, vector-matrix multiplication^5^, quantum information processing^6^, and neural networks^7^. Reconfigurable and programmable PICs usually consist of matrices of integrated photonic devices, such as Mach-Zehnder Interferometers (MZIs)^8^, whose working point can be set on demand to perform dynamic spatial light routing and define the overall photonic functionality^9^. A dedicated electronic control layer is thus needed to enable run-time configuration of these PICs, ideally without requiring any prior calibration. This latter requirement can be achieved by closing around each photonic device a feedback control loop, which assesses the working point in real-time and counteracts unwanted variations from the target. However, as PICs increase in complexity, eventually featuring hundreds of optical devices, the scalability of the control hardware becomes a critical issue. Indeed, currently available discrete components-based electronic boards^10^ do not represent a viable option due to their bulkiness and power consumption, which make them suitable for laboratory demonstrations but unpractical in commercial applications. Such solutions often rely on field programmable gate array (FPGA) controllers, that guarantee great programmability but do not allow single-chip integration of fully functional control loops, comprising sensors readout, analog-to-digital converters and actuators drivers. All this circuitry is added as off-the-shelf discrete components, limiting power and area efficiency of the control system.

**Fig. 1:**
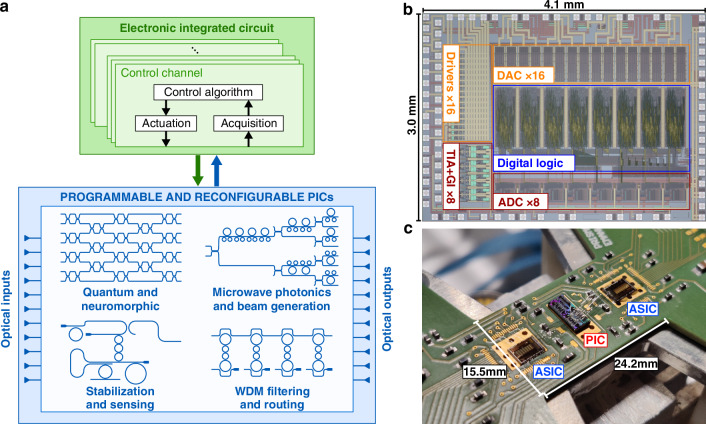
Integrated electronic controller for reconfigurable photonic circuits. **a** Schematic view of some examples of programmable photonic circuits that would greatly benefit from connection to a dedicated electronic control layer, capable of reconfiguring the optical functionality at run-time. **b** Microscope photograph of the custom electronic ASIC for real-time control of programmable PICs described in this work, highlighting its size and main internal sections. **c** Connection of two ASICs to a programmable photonic circuit, demonstrating the compact size of the complete assembly that ensures scalability to large-scale optical architectures

Application-specific integrated circuits (ASICs) offer a promising alternative, as their intrinsic scalability and modularity allow to drive each photonic device independently, consuming only a fraction of the power and area required by an electronic board. Solutions have already been proposed to implement integrated local feedback loops that stabilize the working point of PICs containing few devices against thermal drifts^11,12^; what is still missing is a CMOS architecture that seamlessly extends this approach to larger optical processors, featuring tens of devices, and enables dynamic reconfiguration of PICs, thus opening applications requiring more than the sheer compensation of slow time-varying drifts. To some extent, the “application-specific” paradigm has to be moved from photonic to electronic circuits^1^, with little to no loss in terms of programmability of the optical processor, as conceptually shown in Fig. 1a. A secondary motivation behind the adoption of ASIC controllers can be found considering the fabrication flow involved in integrated optics, which largely relies on long-established processes already developed for CMOS electronic integrated circuits^13^: it is straightforward to think of a chiplet-like electronic-photonic co-design, where the same package contains both ASICs and PICs, which can even be arranged in a flip-chip architecture^14^. Monolithic integration of electrical and optical functionalities on a single silicon die has also been proposed^15,16^, although it still comes with tradeoffs between performance and cost.

To fill this missing gap, in this work we present an 8-channel electronic application-specific integrated circuit (ASIC) designed to perform real-time configuration of programmable photonic chips, requiring no prior calibration to set and maintain the optical functionality effectively. To validate the approach, a pair of ASICs was used to control a 16-channel self-aligning universal beam coupler^17,18^ fabricated in Silicon Photonics technology. Experiments were conducted to assess the ability of the CMOS controller to automatically determine the optimal set of working points of each optical device that maximizes the coupling efficiency between the programmable PIC and the input beam. Time-varying effects affecting the optical link, including dynamic perturbations of the wavefront, were successfully compensated, eventually enabling 25 Gbaud communication through an indoor free-space setup. The proposed ASIC, with a power consumption lower than 10 mW per channel and an overall area occupation of 12 mm^2^, thus represents a key element for supporting scalable and reliable use of photonic integrated circuits in all those applications where static calibrations and lookup tables are not sufficient, such as all-optical signal processors, general purpose programmable PICs and adaptive and reconfigurable devices.

## Results

### Architecture of the integrated CMOS controller

The 8-channel CMOS electronic integrated circuit has been specifically designed for dynamically controlling programmable optical circuits implemented in Silicon Photonics technology. The ASIC has been conceived to operate PICs whose functionality is defined by completely switching on/off optical interconnections, implemented with Mach-Zehnder interferometers. When dealing with MZIs, each channel of the controller simultaneously drives two thermo-optical actuators; however, the chip can also be effectively employed to control microring resonator (MRR) based circuits, where only one actuator is needed, as well as combinations of MZI and MRR and any other photonic circuit. Figure 1b shows a microscope photograph of the CMOS controller. The ASIC, built in AMS CMOS 0.35 *μ*m technology, occupies an active silicon area of ≈12 mm^2^, thus comparable to the photonic chip. The power dissipation of each electronic channel, working with 3.3 V power supply, is ≈ 10 mW, less than what a single thermal actuator requires to achieve a phase shift larger than 2*π*, in the order of ≈30 mW (measurements are provided in Supplementary Section 4). Given the compact size of the electronic chip, multiple ASICs can be connected to a single PIC whenever more than 8 channels have to be managed in parallel. Figure 1c shows the connection between two ASICs and a PIC (described in detail in the following section), made through direct wire bonding to minimize the effect of stray capacitance, maximize the readout accuracy and limit the assembly size. A custom printed circuit board (PCB) has been designed to host the chips during the experimental validation, route the electrical signals necessary for their operation and communicate with a personal computer to configure and monitor the system.

**Fig. 2:**
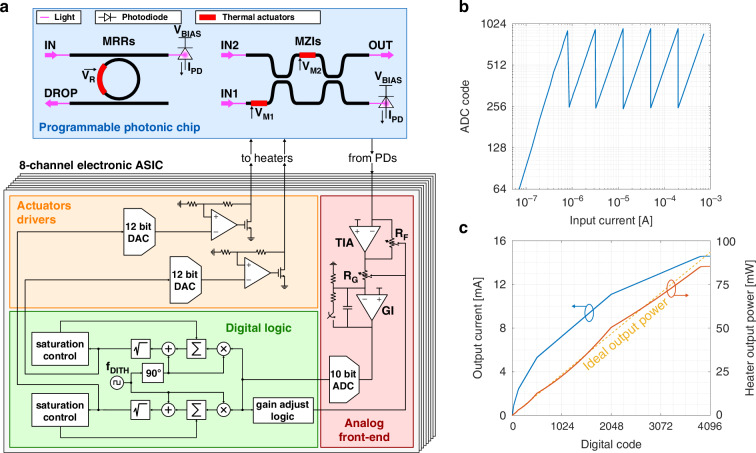
Electronic chip design and characterization. **a** Schematic view of the ASIC architecture for dynamic control of programmable photonic circuits. The ASIC features 8 parallel channels. **b** Measured ADC code for a wide range of input currents, that can be correctly detected thanks to the adaptive amplification mechanism. **c** Characterization of the actuator driver circuit: the square root compression of the DAC output (blue) results in a linearization of the heater dissipated power (red) with respect to the digital control value

The architecture of the electronic control channel is shown in Fig. 2a. The optical power impinging on one output branch of the photonic device is read by an integrated photodiode (PD). In this work, we consider germanium PDs that are commonly provided by foundry process design kits, but other kinds of PDs or monitor photodetector^19^ can be used as well by properly adapting the circuit. The photogenerated current is fed to the analog front-end of the ASIC for amplification through a transimpedance amplifier, low-pass filtering with a gated integrator and digitization by a 10-bit analog-to-digital converter (ADC) working with sampling rate *f*_*s* _= 100 kSamples/s. An input optical power dynamic range of 50 dB has been considered during the design of the analog acquisition chain to correctly detect the photogenerated current (≈30 dB to account for the MZI/MRR rejection ratio, plus an additional margin of 20 dB to adapt to possible coupling losses and different optical power levels). The gain of the front-end stage is automatically selected by a digital logic among 6 different values according to the absolute optical power impinging on the photodetector, in order to always match the ADC full-scale range to the photocurrent level. Optical signals between 0 dBm and −50 dBm can thus be successfully detected and managed by the ASIC, suitable for realistic applications. This is confirmed by the experimental measurement shown in Fig. 2b, which reports the values sampled by the ADC as a function of the input PD current and highlights the operation of the adaptive amplification stage.

The control algorithm to maximize/minimize the optical power at the output of each photonic device, needed to switch on/off the desired optical paths, is implemented at digital level and relies on the dithering technique in combination with integral controllers^20^. This approach allows to define a feedback strategy that is easily scalable to multiple devices and independent of the average light power in the chip and the setup temperature. The error signal minimized by the lock-in based control loop is only related to the partial derivatives of the optical output power with respect to the actuators command: it is thus insensitive to any DC offset, that might result from the sensors dark current or the residual optical power injected from other devices in the PIC (more details on the control algorithm are provided in Supplementary Section 1). The dithering extraction and integration processes are performed in the digital domain, to increase chip flexibility. Two independent chains operating on orthogonal dithering signals are needed when controlling MZIs with two heaters, whereas a single digital circuit is enough for a MRR. Within each chain, a square-wave multiplier downconverts the ADC readout and a digital integrator accumulates the resulting signal, thus simultaneously implementing the integral feedback law needed for controlling the photonic device and precisely defining the control loop bandwidth. The obtained DC signal, upon which the square-wave oscillation is superimposed to apply the dithering modulation to the actuators, is used to update the device working point. A square root operator, whose details are described in Supplementary Section 4, is used to compress the generated digital word and linearize the feedback loop, by compensating the quadratic relation between the heater voltage and its dissipated power. This ensures the same control loop response regardless of the actuators DC bias^14^. The output digital word is finally compared with two programmable thresholds, that trigger the reset of the accumulator at mid-scale in order not to saturate the driving circuit when the generated voltage gets too close to the power supply rails.

The output of the digital logic is fed to a mixed-signal actuation circuit, made of a pair of 12-bit digital-to-analog converters (DACs) and high-current analog drivers. The drivers are designed to operate the integrated thermal phase shifters, which have a nominal resistance *R*_*H*_ ≈ 400 *Ω*, within a 0–6 V range, corresponding to a maximum phase shift of ≈4*π*. In this way, the control loop is always able to find a minimum in the device transfer function regardless of the algorithm starting condition. The resolution of the DACs has been chosen to achieve an accuracy of ≈ 1.5 mV in the generation of the actuators voltage, enough to perform an effective control action. Figure 2c shows the measured current provided by the driving stage to the actuators, and the corresponding heater dissipated power, confirming the effective linearization performed by the square root operator and the sufficient power capability of the circuit.

### Integrated 16-channel self-aligning beam coupler

The designed ASICs have been used to dynamically control a 16-channel silicon photonics integrated self-aligning universal beam coupler (UBC). UBCs are a class of reconfigurable PICs that can receive an arbitrary monochromatic input beam and couple it to a single-mode waveguide^17,18^. They can be implemented as a combination of 2 × 2 interferometers, such as Mach-Zehnder interferometers (MZIs), arranged in a mesh configuration^10,21^. By making each interferometer tunable with proper on-chip actuators, an adaptive UBC can be dynamically reconfigured to work as a coherent intensity adder and constantly maximize the input-output coupling efficiency even when the input beam characteristics change. The reconfigurability of UBCs makes them suitable to track and mitigate the effect of dynamic phenomena, such as time-varying angular misalignments (i.e., direction of arrival) between an input beam and the photonic chip and/or wave-front distortions caused by propagation through aberrators, scattering media, multimode or multicore fibers/waveguides or turbulent environments, as in the case of atmospheric turbulence in free-space optical (FSO) links^22^.

**Fig. 3:**
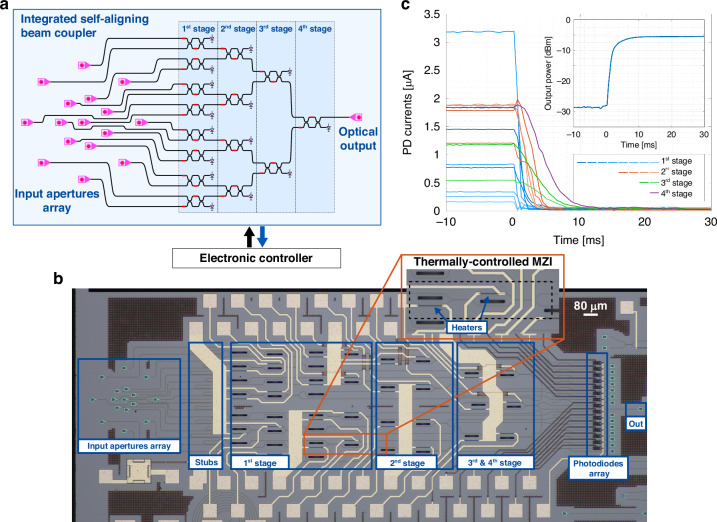
Silicon photonics 16-channel self-aligning universal beam coupler. **a** Schematic view and **b** microscope photograph of the self-configuring optical beam coupler, made of a binary-tree mesh of 15 MZIs. A detail of a thermally-tunable MZI, featuring two actuators to completely steer the input light to one of the device outputs, is also reported. **c** Time transient of the PDs photocurrents when configuring the optical circuit, showing correct minimization when the ASICs are activated after 10 ms. The inset shows the evolution of the chip output power, which is correspondingly maximized in around 10 ms

A schematic of the designed integrated photonic circuit and its top-view microscope photograph are shown in Fig. 3a, b. The circuit has been manufactured by a commercial silicon photonic foundry (Advanced Micro Foundry, Singapore) and has an area of 3.2 mm × 1.3 mm. It consists of 15 thermally-tunable balanced MZIs arranged in a 4-stage 16 × 1 binary mesh. Compared to other mesh topologies^17,21^, this arrangement equalizes the optical path from all inputs to the output and minimizes the number of cascaded MZI stages, thus resulting in a faster configuration time for the receiver. At the input (left-hand side in Fig. 3a), the MZI mesh is connected to an integrated 16-element optical antenna array (OAA). The antennas are realized by using surface grating couplers (GCs) designed to operate on transverse-electric (TE) polarized light. The 16 GCs are arranged in two concentric rings with a radius of 60 *μ*m (inner ring, 7 GCs) and 180 *μ*m (outer ring, 8 GCs), respectively, and a central GC, as shown in Fig. 3b. This OAA configuration is optimized to receive optical beams with a linear polarization and circular symmetry, but other OAA topologies can be designed for other classes of beams^23^. Waveguide stubs are inserted between the OAA and the MZI mesh to match the length of each input optical path and minimize the wavelength dependence of the UBC^21^ (see Methods for further details). At the output of the MZI mesh, 15 waveguides are coupled to integrated photodetectors, while the 16^*th*^ waveguide (optical output) is terminated with a GC for coupling with an optical fiber.

Two ASICs have been used to control the UBC. To demonstrate the possibility of configuring the photonic circuit starting from any initial condition, heater voltages have been initially set to random values; then, the 16 control loops have been simultaneously activated. Figure 3c shows the measured PD currents during the configuration transient of the photonic circuit. All photocurrents are minimized by the ASICs, thus steering all the input light towards the chip optical output. The configuration of the first stage of the receiver takes place in around 1.5 ms, while the following layers are characterized by a slightly longer convergence time. In fact, the *N*^*th*^ stage of the mesh cannot be completely tuned before all the previous *N*−1 stages have been set. This is visible in the figure, which highlights the sequential tuning of the circuit. Even with 4 cascaded stages, the electronic ASICs can configure the whole MZI architecture in around 10 ms, as shown by the transient of the optical output power in the inset of the figure. It is worth pointing out that all MZIs are configured by using the same dithering frequency. This approach simplifies the control electronics and does not prevent correct configuration of the PIC. Indeed, once the MZIs of the *N*^*th*^ stage are locked to the minima/maxima of their transfer function, they do not inject any disturbance to the *N*^*th*^ + 1 stage, since the residual dithering oscillation at their output is nil in stationary points.

### Dynamic correction of wavefront distortions

**Fig. 4:**
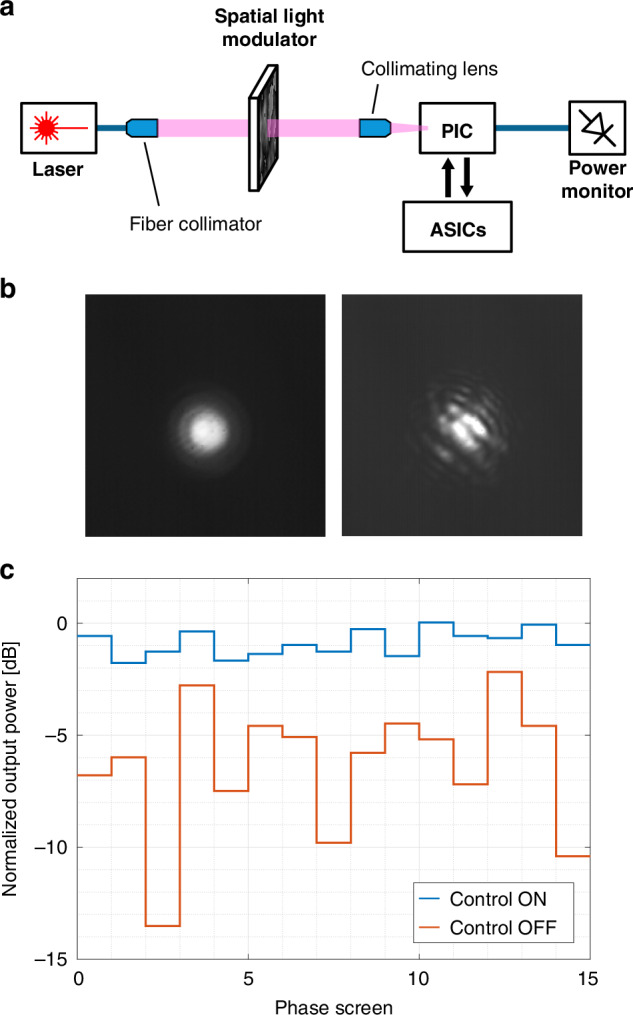
Experimental validation. **a** Schematic view of the setup employed to introduce a static perturbation in the wavefront of a free-space beam. **b** Photograph, captured with an infrared camera, of the optical beam impinging on the beam coupler, when the SLM is off (left) and when it is used to perturb the free-space beam (right). **c** Optical power at the output port of the beam coupler for different SLM phase screens, showing its ability to perform real-time wavefront correction and beam reconstruction

The performance of the ASIC controller was assessed by testing the UBC as a dynamic tracker of wavefront distortions of free-space optical beams. In the first experiment (Fig. 4a), static distortions with a well-controllable profile were intentionally introduced by means of a spatial light modulator (SLM) placed along the propagation path of the beam (see Methods and Supplementary Section 2 for details on the experimental setup). The SLM was used to generate 15 synthetic phase masks (reported in Supplementary Section 3) and an infrared camera was used to capture the resulting perturbation effects. As reported in Fig. 4b, the Gaussian profile of the input beam (left panel) is significantly altered after reflection on the SLM, with a significant change of shape, size, and centroid (right panel). The distorted beam was coupled to the OAA of the PIC through a collimating lens. The performance of the ASIC-controlled PIC was then assessed by measuring the optical power at the output port of the UBC when the ASIC control is switched off (all the heaters are held at a constant voltage) and when the feedback control is activated to find the best mesh configuration and maximize the output power. Figure 4c shows the optical output power for different phase screens of the SLM (data are normalized to the output power received when SLM is off and no perturbation is introduced). When the control is turned off, the average received power is −5.7 dB with a standard deviation of 2.9 dB. Instead, when the UBC is automatically reconfigured after changing the SLM mask, the average power improves to −0.8 dB and the standard deviation reduces to 0.6 dB, thus demonstrating the effectiveness of the adaptive ASIC controller. The slight residual fading is due to the shift of the centroid of the beam causing variations of the total power impinging on the GCs of the OAA under different perturbation profiles. This can be improved with a larger OAA and with a higher fill factor, obtained by employing more GCs, an array of lenslets or a photonic lantern device^21^.

**Fig. 5:**
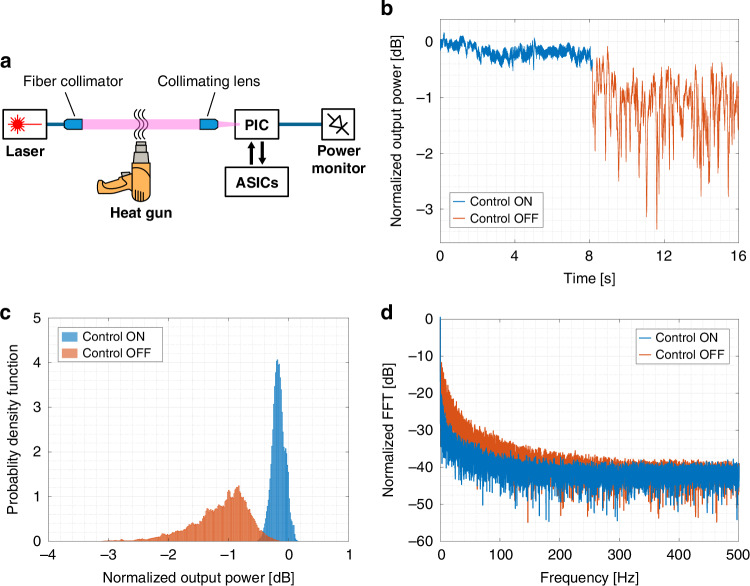
Real-time compensation of dynamic perturbations. **a** Experimental setup employed to introduce a dynamic perturbation in the free-space propagation of the beam. **b** Temporal evolution, **c** probability density function, and **d** frequency spectrum of the received optical power when the ASICs are active (blue curves) or disabled (orange curves), demonstrating correct real-time compensation of the beam-front distortion performed by the electronically controlled photonic chip

To test the ASIC controller in dynamic conditions with fast wavefront distortions of the optical beam, the SLM was replaced with a heat gun introducing turbulence in the free space path (Fig. 5a). Figure 5b shows the measured power at the optical output of the PIC when the control ASICs are activated (blue curve) / deactivated (orange curve) (data are normalized to the output power received when the PIC is configured for an unperturbed Gaussian beam, obtained with the heat gun turned off). When the ASICs are on, the UBC compensates for the wavefront distortion and the detected optical power at the output has a mean value of −0.17 dB, a standard deviation of 0.1 dB and a maximum fading of less than 0.5 dB. After 8 s, the control loops are paused, by holding the heater driving voltages at fixed values. In this condition, the PIC is on average well configured to receive the incoming beam, however the random phase front fluctuations result in a degradation of the received power: a mean value of −1.15 dB and a standard deviation of 0.5 dB are observed, with a maximum fading higher than 3 dB. The same is clearly visible in Fig. 5c, which compares the probability density function of the received optical power in the two situations. These results confirm the advantage of dynamic and real-time PIC reconfiguration, which translates in a narrower distribution concentrated at higher power levels: 90% of the samples are above the threshold of −0.3 dB when the controller is tracking the injected turbulence, whereas the same parameter worsens to −1.83 dB when no control is exerted (and only ≈ 1% of the samples stay above −0.3 dB). Figure 5d shows the frequency spectrum of the optical signal at the output waveguide of the UBC. When the control loops are off (orange curve), spectral components extending up to about 300 Hz are observed, which are related to the harmonics of the perturbation generated by the heat gun. The spectrum acquired after the activation of the ASIC controllers (blue curve) confirms that the real-time reconfiguration of the PIC significantly reduces the perturbation effect on the output up to around 300 Hz. This demonstrates that the tracking time of the ASIC-controlled PIC is faster than 10 ms. Notably, this response time is faster than the dynamics of atmospheric turbulence^24^, meaning that our approach can be effectively used to compensate for wavefront distortion in real free space links for communication, sensing and ranging applications.

### ASIC-assisted adaptive free-space optical receiver

**Fig. 6:**
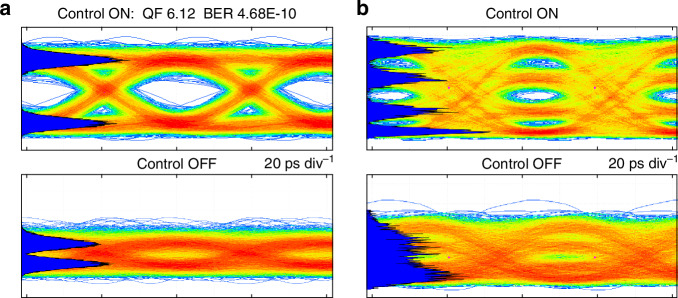
High-speed free-space optical transmission. Eye diagrams of the received optical signal when transmitting a free-space beam modulated at 25 Gbaud both with **a** OOK and **b** PAM4 modulations, showing the transmission improvement when the adaptive receiver is dynamically operated

The ASIC-assisted UBC was finally employed to receive a high-data-rate free-space optical signal corrupted by wavefront distortions. To this aim, the laser beam was modulated in intensity at a symbol rate of 25 Gbaud with a commercial modulator and transmitted through a free-space optical link. As in previous experiments, at the receiver side the optical beam was coupled to the OAA of the UBC, whose optical output was captured with a single-mode optical fiber and monitored with a real-time optical oscilloscope to assess the signal quality. No signal equalization has been performed. The results of the data transmission experiment are shown in Fig. 6a, for the case of 25 Gbit/s non-return-to-zero on-off keying modulation. When the working point of the photonic processor is not optimized by the ASICs (bottom panel), the input beams sampled by the OAA are combined along the MZI mesh with random phase relations, resulting in a degraded signal with a completely closed eye diagram at the chip output. In contrast, a fully open eye diagram with a quality factor of 6.12 is recorded when the ASIC controllers are activated. Notably, the control of the coherent adder performed by the ASIC controller is independent of the modulation format and data rate of the transmitted signal. Figure 6b shows the eye diagrams of a 50 Gbit/s 4-level pulse-amplitude modulation (PAM-4) with (upper panel) and without (lower panel) adaptive PIC configuration, confirming a clear improvement of the quality of the received signal when the ASIC controller is activated.

## Discussion

We demonstrated dynamic reconfiguration, control and tracking of PICs made of many tunable elements by using parallel feedback loops integrated in a single multi-channel ASIC controller. The ASIC can automatically set the working point of the photonic chip in real-time and lock the photonic functionality to the desired one. The compact size (similar to a PIC) and low power consumption (the entire electronic channel dissipates less power than a single heater) make the ASIC a pivotal solution for scaling the complexity of programmable PICs to several tens of devices, a level that can be hardly managed with off-the-shelf solutions. As an example of application, we validated the use of two 8-channel ASICs to dynamically operate a 16-channel thermally tunable MZI silicon photonics mesh as an adaptive coherent adder. The ASIC controller enables both automated configuration of the programmable PIC and effective compensation of dynamic wavefront distortions of the input beam, with no need for any prior calibration nor knowledge of the turbulent transmission channel. The combination of ASICs and PIC allowed us to automatically couple a free-space beam to a single-mode waveguide even in the presence of dynamic wavefront perturbations, thanks to a control bandwidth around 400 Hz. Successful reconstruction of a 50 Gbit/s PAM-4 modulated signal demonstrated the correct operation of the electronic-assisted UBC, validating the operation of the electronic controller.

To the best of the authors’ knowledge, the ASIC represents the first demonstration of fully-integrated electronic controller for programmable photonic circuits. Discrete-components solutions based on commercial digital signal processors exist, ensuring great flexibility in the design of the feedback algorithm^25,26^. They are thus perfectly suited for prototype implementations, where the photonic circuit to be operated changes depending on the particular application. However, these controllers are not a viable solution when scaling beyond laboratory environment, due to unavoidable limitations in terms of area, power dissipation and cost. To overcome these limitations, partial integration of the control circuit was proposed^12,15^, simplifying the external electronics to fewer components or even to only the digital processing core. These hybrid options allow the validation and optimization of different control algorithms in a relatively compact footprint. However, they are still not competitive with fully-integrated control chips when very efficient systems-in-package are targeted. This is the case, for example, of the circuits reported in^11,14^, which focus on optical transceivers based on MRRs. Our ASIC extends the control paradigm also to the more complex case of MZIs, thus providing a more general platform suitable for reconfigurable PICs. Supplementary Section 6 reports a detailed comparison among electronic controllers for PIC reconfiguration, highlighting the strengths of the proposed approach.

UBCs are one example of reconfigurable photonic circuits that can be controlled by the presented ASIC. However, the chip can operate any PIC that is programmed by completely switching on/off local optical paths. This applies to circuits based on both MZIs and MRRs, since the feedback logic to minimize or maximize light is independent of the particular photonic device of interest. The ASIC could thus be readily used to control optical routers and distribution networks^27,28^, wavelength-selective filters and (de)multiplexers^29,30^, as well as beam formers and analyzers^31,32^. Other applications, instead, require partial light splitting among different optical paths. This is the case, for example, of vector-matrix multiplication circuits^5^, mode unscramblers^4^ and optical neural networks^33^. In these situations, a different control strategy needs to be implemented^34^, requiring an update of the ASIC digital structure. With this simple adaptation, the chip would be beneficial even in these applications, thanks to its limited area and power consumption. In addition, the use of minimally-invasive PDs^19^ can be particularly attractive in certain photonic circuits, since they can be placed in-line with the optical path without significant penalties. Addressing these devices, which further increase the programmability of PICs, would only require minimal corrections in the ASIC front-end, without any modification of the control algorithm. These improvements would make control ASICs versatile and suitable for a wider range of applications and reconfigurable PICs, further boosting its pivotal role.

Technological improvements in the ASIC manufacturing and packaging are another possible evolution of this work. A more advanced electronic technology node can be used to fabricate the ASIC, significantly reducing the area of the single control channel and thus allowing the integration of more feedback loops on the same die. This simplifies the assembly and packaging procedure when large-scale PICs need to be operated, improving also the reliability of the whole system. The ultimate goal is to design a control chain with an area occupation comparable to a single photonic device, allowing one-to-one matching between optical and electronic footprints. With this approach, the ASIC could be connected in a flip-chip arrangement to the PIC, acting both as optical element and mechanical substrate^35^. The result can be achieved with mature, long-established sub-100 nm nodes^14^, although designing a 3D-integrated controller enabling full PIC programmability beyond thermal drift compensation has not been demonstrated yet. Flip-chip integration also addresses one scalability limit that the proposed system still faces, i.e., the density of connections. Each optical device requires 2 (MRRs) or 3 (MZIs) connections to close the corresponding electronic control loop: despite the good level of compactness, wire-bonding is a solution that cannot scale indefinitely; flip-chip connections, instead, break the shoreline limitation, provided that the ASIC is shrunk down to the size of its optical counterpart. While ensuring higher integration density and reduction of parasitics and crosstalk, flip-chip solutions are more critical in terms of packaging and, most importantly, thermal management^35^, requiring careful system design optimization.

The general need for reduced power consumption also advocates for the adoption of a more scaled technology node and possibly non-thermal actuators. As the dissipation of the digital blocks scales with the transistor size, smaller devices would improve the efficiency of the chip. A more advanced node would also improve the interface circuits. As an example, we could implement the readout circuit with an oversampling ADC, achieving better resolution at reasonable speed (typically beyond 14 bits in the audio frequency range^36^) and inherent anti-aliasing filtering action. The former improvement would ease the complexity of the adaptive amplification stage, while the latter would allow us to remove the analog gated integrator. Similarly, scaled transistors enable the design of more advanced actuator driving stages, for example those employing pulse width modulation (PWM)^28^, that would also contribute to reducing the chip power consumption. When driving heaters with such techniques, the PWM command frequency easily scales beyond 1 MHz to minimize the residual thermal ripple^37^, meaning that a 12-bit DAC should discriminate duty cycles with on-times that differ by some hundreds of ps. A scaled technology, with reduced propagation delays, surely would help. With these improvements, the ASIC becomes attractive also when non-thermal actuators with high energy efficiency (e.g., devices exploiting materials with high electro-optic coefficients or opto-mechanical phase shifters^38,39^) are employed, even though the presented approach is valid regardless of the actuators technology of choice.

## Methods

### ASIC fabrication and characterization

The CMOS chip has been designed in order to read and control the operating point of each photonic device with proper accuracy, considering a maximum rejection of ≈ 30 dB between the output ports. An optical power impinging on the PD ranging from 0 dBm to −20 dBm has also been considered, therefore the circuit must be able to sense optical power variations as small as −50 dBm. In principle, this would require a complex 16-bit ADC; instead, a simple 10-bit converter has been employed. The required precision has been achieved by automatically adjusting the analog gain of the acquisition chain according to the average impinging optical power, which is sampled every time the working points of the heaters are updated: whenever it scales by a factor 4, the amplification implemented by the analog readout is increased accordingly (as demonstrated by the log-log plot of Fig. 2b), thus always guaranteeing a rail-to-rail mapping of the given current range. At the same time, when the analog gain doubles, the weight of the ADC samples fed to the digital accumulator is halved, keeping the demodulation process coherent with the decreasing optical power.

Concerning the actuators, DACs with 12-bit resolution have been employed to ensure sufficient precision in controlling the operating point of the heaters, as well as to provide a wide variety of dithering modulation amplitudes, usually in the 3–50 mV range. The DACs have been designed with monotonic behavior, which guarantees that the loop sign remains unchanged during operations and preserves the control stability. Improved linearity in the control response has been obtained thanks to the digital square root compression performed before the DACs. The operation is implemented by approximating the exact square root calculation with a piece-wise linear function, whose slope is halved every time its digital input is increased by a factor of 4. The solution requires fewer resources compared to more precise digital architectures^40^ and offers a wider operating range compared to analog solutions^14^.

Finally, the design features a pair of shift registers for interfacing the chip with a personal computer: one is used to set the working parameters of the circuit, such as the control bandwidth, and to manually configure the operating point of the PIC, which can be useful when characterizing the optical devices; the other monitors the photogenerated current of each PD, as sampled by the ADC, and the voltage applied to each thermal actuator. These registers allow easy electrical characterization of the ASIC, which is available in Supplementary Section 4.

### Photonic chip design and fabrication

The integrated photonic processor has been designed for operation around the 1550 nm wavelength range and fabricated on a standard 220 nm Silicon Photonics platform. The photonic chip size is 3.2 mm × 1.3 mm. All the waveguides of the circuit are single-mode channel waveguides with a propagation loss of about 1 dB/cm, resulting in a ≈ 2 dB loss in the end-to-end path across the PIC. The waveguides connecting the grating couplers to the photonic processor have the same geometrical length to minimize the wavelength dependence of the mesh, so that the circuit can have the widest optical bandwidth. Each GC is about 23 *μ*m-wide and 48 *μ*m-long (including a 24 *μ*m-long taper). The elevation angle of the radiated field is 12^∘^, the azimuth angle is 0^∘^, and the divergence is 5.6^∘^ × 9.8^∘^, resulting in a coupling loss of the single GC of about 4.5 dB. When receiving a free-space beam, an additional geometric loss of around 20 dB must be considered because of the limited fill factor of the optical antennas. This latter loss could be reduced by increasing the size of the mesh: doubling the number of apertures, for example, would halve the geometric loss, at the price of a larger PIC footprint.

The MZIs have been designed with 3 dB directional couplers and two TiN thermal tuners, and feature a maximum extinction ratio of about 25 dB. The heaters are placed on top of the MZI input waveguide and on one of the internal interferometer arms. This enables the control of the relative phase shift between the optical fields at the MZI input ports and the amplitude split ratio of the interferometer, respectively. The thermal tuners have a power efficiency of 22 mW/*π* and a time response of about 10 *μ*s. The maximum power consumption of the photonic processor is about 750 mW in the worst case, i.e., when a 2*π* phase shift needs to be simultaneously applied to all the thermal tuners. The scalability of the system to larger circuits would benefit from the use of non-thermal actuators, exploiting materials with high electro-optic coefficients such as lithium niobate or barium titanate integrated in silicon waveguides, or opto-micromechanical phase shifters. More details on the main photonic building blocks employed in the design of the UBC are reported in Supplementary Section 5.

The wavelength range across which the photonic processors can operate is about 35 nm (from 1535 nm to 1570 nm)^21^. This range is mainly limited by the wavelength-selective response of the GCs and by the wavelength dependence of the 3 dB directional couplers of the MZIs. Such wavelength dependence can be reduced by using optimized designs for broadband GCs^41^ and directional couplers^42^. The broadband operation of the UBC thus enables simultaneous reception of multiple wavelength-division-multiplexed channels, providing an effective way for scaling the capacity of FSO links.

### Experimental setup

The dynamic control of the ASICs has been experimentally tested on a 3 m-long indoor free-space optical link. A laser with 1550 nm wavelength and 5 dBm output power, amplified by 20 dB with a commercial erbium-doped fiber amplifier, has been used in the experiments. The laser output has been launched into free space with a fiber collimator and then expanded to a diameter of around 5 cm with a set of lenses. Intentional distortions in the beam wavefront have been introduced with a spatial light modulator (SLM) and a thermal gun along the propagation path, thus requiring a continuous update of the UBC working point to enable correct signal reception. The beam has been coupled to the photonic chip with an optical system consisting of a 45^∘^-tilted dielectric mirror and a set of collimating lenses. The coherently recombined signal of the last MZI has been coupled out of the chip with a vertically aligned single-mode fiber and measured with bench-top instruments. The temperature of the assembly has been measured with a thermistor placed next to the photonic chip and the whole setup has been kept at 28 ^∘^C with a thermo-electric cooler (TEC). More details on the optical setup are reported in Supplementary Section 2.

## Supplementary information

Integrated electronic controller for dynamic self-configuration of photonic circuits - Supplementary material

## Data Availability

All the data supporting the findings of this study are available within this Article and its Supplementary Information. Any additional data are available from the corresponding author upon reasonable request.
