# Supplementary material for: Integrated electronic controller for dynamic self-configuration of photonic circuits
Source: Light Sci Appl. 2025 Sep 30;14:348. doi: 10.1038/s41377-025-01977-w (PMC12480261; doi:10.1038/s41377-025-01977-w)
Supplement: Supplementary file 1 — Integrated electronic controller for dynamic self-configuration of photonic circuits - Supplementary material [file 41377_2025_1977_MOESM1_ESM.pdf]

Integrated electronic controller for dynamic self-configuration of photonic circuits

Supplementary material

Emanuele Sacchi[1], Francesco Zanetto[1], Andrés Ivan Martinez[1], SeyedMohammad SeyedinNavadeh[1], Francesco Morichetti[1], Andrea Melloni[1], Marco Sampietro[1] and Giorgio Ferrari[2]

[1]Department of Electronics, Information and Bioengineering, Politecnico di Milano, piazza Leonardo da Vinci 32, Milano, 20133, Italy.
[2]Department of Physics, Politecnico di Milano, piazza Leonardo da Vinci 32, Milano, 20133, Italy.

Corresponding author: emanuele.sacchi@polimi.it;

Supplementary section 1: dynamic self-configuration of universal beam couplers

The proposed control strategy for automatically configuring each photonic device relies on the dithering technique to realize a calibration-free feedback loop that does not require prior knowledge of the device transfer function. Here, the main concepts of this technique are briefly summarized, while more details on its implementation can be found in [1, 2].

Figure 1a shows the working principle of the dithering technique applied to a single thermally tunable Mach-Zehnder interferometer (MZI). A small modulation is superimposed on the DC voltage of each actuator (H1, H2), making the device transfer function oscillate around its bias point with a modulation depth proportional to the transfer function first derivative. By choosing orthogonal (e.g. in-phase/quadrature) dithering signals, the effect of the two MZI actuators can be discriminated with a lock-in demodulation of the signal provided by a single photodetector placed at the drop port [3]. In this way, the MZI transfer function and its partial derivatives with respect to the heaters power can be extracted in real time from the physical system.

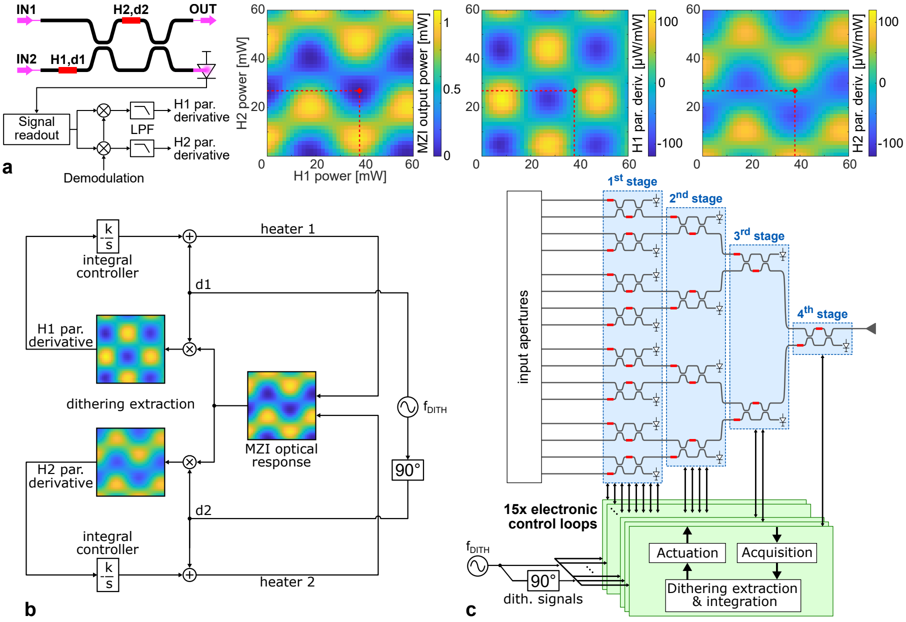

Fig. 1 Automated control of self-aligning beam couplers. a) Working principle and experimental demonstration of the dithering technique, that allows extracting in real-time the partial derivatives of the MZI transfer function with respect to the electrical power dissipated by the heaters (H1, H2) in addition to the absolute output optical power. b) Schematic view of the real-time control scheme applied to a single MZI, allowing automated minimization of the light reaching the photodiodes. c) Seamless extension of the feedback approach to a multi-aperture self-aligning beam coupler, only requiring two dithering signals to control multiple interferometers.

The information on the partial derivatives is then exploited to control the MZI and minimize the amount of light detected at the drop port. This is obtained by tuning the DC heater voltages to drive the output dithering signals to zero, as this condition corresponds to a stationary point of the MZI transfer function. With respect to other techniques [4–7], this approach does not require any prior calibration, since the condition of null output dithering oscillation only depends on the MZI transfer function and is not affected by any other parameter of the system, such as instabilities of the input light power or temperature drifts. The DC heater voltage is automatically set by two parallel integral controllers, each fed with one of the two partial derivative signals (Figure 1b). The sign of the integral controller gain is adjusted to provide a negative feedback loop only when the minima of the MZI transfer function are reached, thus avoiding ambiguity on the target locking point.

The technique can be straightforwardly extended to control a more complex photonic circuit such as a multi-aperture beam coupler, by implementing multiple identical and independent control loops, as shown in Figure 1c for the case of a binary mesh with 15 MZIs. Thanks to the particular structure of the photonic circuit, requiring sequential configuration of each stage of the mesh, all the control loops can exploit the same dithering signals [1]. Indeed, once the MZIs of a certain stage of the receiver are successfully configured, the residual dithering oscillation at their output is zeroed and does not affect the configuration of the following devices. The interaction between stages only happens during an initial transitory phase and it does not impair the correct operation of the mesh. This approach simplifies the requirements of the control electronics, since only 2 dithering signals are necessary to control 30 heaters and a single-frequency lock-in readout is sufficient to implement an effective control action on the whole photonic circuit.

Once the mesh configuration is completed, the control loops are kept active to counteract dynamic perturbation effects in real-time, such as turbulence-induced amplitude and phase fluctuations of the incoming optical beam and thermal variations. Interestingly, the bandwidth (BW) of the control system is defined by the response time of the single MZI (time constant of 400 µs, corresponding to a BW around 400 Hz in our case) and not of the full receiver. Indeed, if the perturbation is within the bandwidth of the single MZI control, each feedback loop is able to constantly update the heaters voltage and keep the interferometers in the correct operating point, thus avoiding interactions between the stages of the mesh. Therefore, the chip can successfully counteract the effect of realistic turbulence effects, as shown in the experiments.

It is worth pointing out that, when using the dithering technique, the light modulation at each MZI output is proportional to both the device transfer function first derivative and the injected oscillation amplitude. Therefore, the response time of the control loop is affected by this parameter. This is shown in Figure 2, which details the time evolution of a single MZI control when, starting from the same initial condition, the feedback is activated with different dithering amplitudes. The 90-10% transient ranges from around 1.5 ms, when

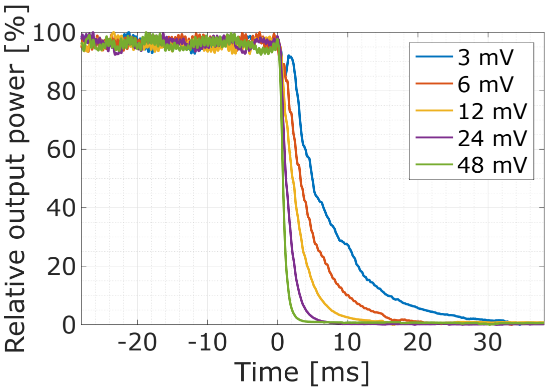

Fig. 2 Impact of different dithering amplitudes. Time traces of the optical power measured by the integrated photodiode of a single MZI when activating the control loop (at t=0 ms). After each measurement, the initial working point of the interferometer is reset at the same value, demonstrating the impact of using different dithering amplitudes on the transient response of the device.

a 48 mV modulation is applied (equivalent to 0.8% of the actuators power supply), to more than 20 ms when we only modulate 0.5‰ of the available full-scale range. Increasing the dithering amplitude is thus desirable to improve the control loop performance. However, the oscillation should be small enough not to significantly perturb the optical performance of the MZI. For this reason, we limited it to a maximum value of 48 mV.

Once the dithering amplitude is selected, two other considerations should be made in order to define the feedback loop bandwidth by setting the gain k of the integral controller. First, there is an unavoidable trade-off between the response time and the actuation noise: a faster feedback loop results in a less accurate heater voltage generated by the controller, as reported in [1]. Therefore, once the noise sources in the system are properly minimized with a correct electronic design, the control bandwidth must be selected by properly balancing the desired response time and accuracy. We selected a bandwidth of around 400 Hz since it is wide enough to mitigate environmental perturbations while resulting in a heater command noise well below the amplitude of the dithering oscillation. In this way, the accuracy of the control action is not impaired. An additional upper limit to the bandwidth also exists, for at least one dithering modulation cycle is needed to correctly assess the working point of a photonic device. We thus set the dithering frequency to 8 kHz, fast enough to achieve the desired control bandwidth but easily detected with a relatively slow ADC.

Supplementary section 2: experimental free-space setup

The experimental setup employed in the experiments presented in this work is shown in Supplementary Figure 3. It has been conceived to emulate an outdoor medium-distance link in an indoor controlled environment, thus easing system-level testing.

The laser source (wavelength centered at 1550 nm) and the optical modulator are integrated into the ID Photonics IQ Optical Multi-Format Transmitter. The 25 Gbaud on-off keying (OOK) and 4-level pulse amplitude modulation (PAM4) radio-frequency signals are generated using the Multilane ML4039EN BERT. The resulting optical signal is amplified by an erbium-doped fiber amplifier (EDFA, IPG Photonics EAD-1K-C) up to a power level of 25 dBm before being coupled to a fiber collimator. Such an EDFA is used to compensate for the insertion loss of the modulator and the coupling losses between the FSO beam and the input apertures (2D array of grating couplers) and at the chip output port. The amplified spontaneous emission (ASE) noise of the EDFA is reduced with an ASE rejection filter, with a -3 dB bandwidth of 0.25 nm centered around the carrier wavelength. A fiber polarization controller is used to align the polarization state of the light at the chip input to the transverse electric (TE) mode of the grating coupler.

The FSO beam is generated with a fiber collimator coupled to a free-space beam expander, made of a biconcave lens (LD2060-C) and a biconvex lens (LB1630-C) with a maximization of 6.6. In this way, a free-space beam with a waist of around 5 cm is generated after a free-space propagation of 3 meters. A folded setup, where the light beam is reflected with a spatial light modulator (SLM) after half its propagation, has been employed to reduce the area occupation of the system. The SLM can also be used to steer the beam between the chip input and a near-infrared camera (Xenics Bobcat 640), which

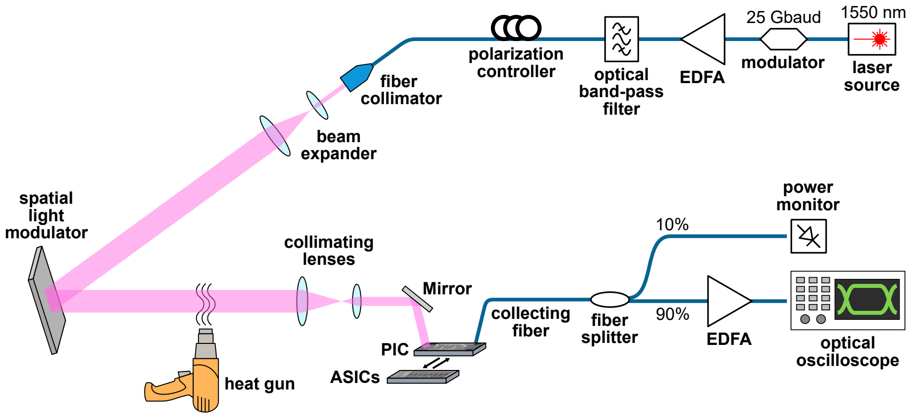

Fig. 3 Experimental setup. Schematic view of the free-space experimental setup for testing the integrated circuits.

is useful for monitoring and characterizing the effect of turbulence. A heat gun is included in the setup for generating dynamic perturbations (in this configuration, the SLM works as a plane mirror and does not introduce any perturbation of the phase profile of the beamfront). By changing the SLM mask or gun position, we could vary the turbulence strength from an effective refractive index parameter C_n^2 of 10^{-14} $m^{-2/3}$ up to 10^{-10} $m^{-2/3}$. The results shown in this article have been obtained by emulating a turbulence strength in the order of 10^{-12} $m^{-2/3}$. Due to the size of the beam and the short length of the link, the wander effect was negligible.

A second set of lenses (LB1889-C f=250 and LD2060-C f=-15) is placed before the chip input to shrink the beam size down to the dimension of the integrated optical antenna array (diameter of around 360 µm). A turning mirror is also positioned on top of the photonic chip to steer the horizontal optical beam to the tilt angle of the on-chip grating couplers (12° with respect to the normal of the chip surface). A single-mode (SM) fiber is positioned on top of the output grating coupler using a 3-axis micro-positioner. A fiber splitter is then used to direct 10% of the optical power towards a bench-top monitor photodetector (HP 81521B), used to assess the system state and characterize the perturbation temporal dynamics. The rest of the light is sent to an EDFA (Amionics EDFA-PA-35-B-FA) to amplify the signal to match the dynamic range of the high-speed optical oscilloscope (Tektronix, DPO75002SX) and evaluate the transmission performance.

The photonic and electronic chips are mounted on a custom-printed circuit board (PCB). The board allows easy electrical routing to the integrated circuits. It provides the power supply to the ASICs, as well as the reference voltages necessary for their operations. It is also needed to interface the chips with a personal computer, for real-time monitoring of the system state. The shape of the PCB has been conceived to allow easy optical access to the photonic chip, both at the free-space input side and at its output with an optical fiber. The temperature of the assembly is monitored with a thermistor placed close to the photonic chip and stabilized to 28 °C using a thermo-electric cooler (TEC), mounted underneath the PCB.

Supplementary section 3: transmission performance with static turbulence

The spatial light modulator (SLM) has been used to introduce static perturbations in the FSO link with a well-defined profile. The phase screens have been properly engineered to simulate free-space propagation for a distance of a few hundred meters, with a perturbation having an effective refractive index parameter C_n^2 of 10^{-12} $\mathrm{m}^{-2/3}$. Supplementary Figure 4 reports the 15 different phase masks employed in the experiment. The electronic-assisted universal

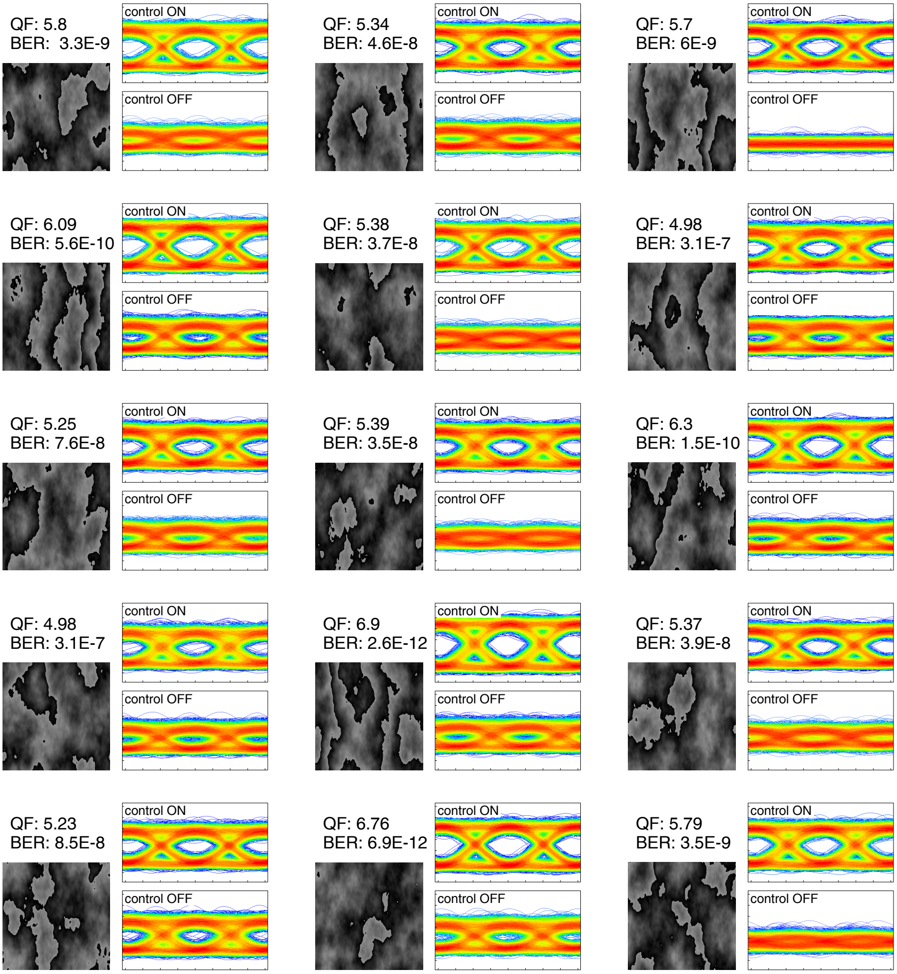

Fig. 4 Generation and mitigation of static turbulence in the FSO link. Picture of the 15 SLM phase screens employed to generate a controlled perturbation in the propagating free-space beam. The corresponding eye diagrams at 25 Gbit/s at the PIC output when the ASICs are on/off are also reported, demonstrating the successful compensation of the perturbation performed by the chips.

beam coupler (UBC) has been used to compensate for the effect of the perturbation. Figure 5c in the main manuscript shows the collected optical power at the chip output, when the ASICs are activated after changing the phase screens and when a static UBC configuration is maintained for all the masks. As expected, a signal with higher average optical power and lower standard deviation is observed when the electronic chips dynamically reconfigure the optical circuit. To further validate this result, we modulated the input light beam with a 25 Gbit/s on-off keying (OOK) non-return-to-zero (NRZ) signal, thus emulating a free-space high-speed communication through a turbulent channel. Supplementary Figure 4 shows the eye diagrams at the PIC output for each SLM mask, when the control loops are on/off. When the turbulence effect is not counteracted by the adaptive receiver, the transmission quality is completely degraded and closed eye diagrams are observed. Instead, the dynamic reconfiguration of the UBC ensures a correct reception of the modulated signal, as confirmed by the acquired eye diagrams that show a quality factor (QF) with an average value of 5.68 and a standard deviation of 0.6. This result confirms the advantage of the adaptive receiver and certifies the correct control action performed by the electronic chips.

Supplementary section 4: electronic chip characterization

The electronic application-specific integrated circuit (ASIC) has been electrically tested to verify the performance of both the front-end readout and actuator driving stages. The schematic of a single ASIC channel is reported in Supplementary Figure 5a for convenience.

The analog front-end, working with a 3.3 V power supply, comprises a transimpedance amplifier (TIA), receiving the photodiode (PD) current, a gated integrator (GI), serving as a low-pass filter, and a 10-bit successive approximation analog-to-digital converter (ADC), chosen to limit area and dissipated power. The ADC is provided as a standard cell by the foundry (AMS, Austria) libraries [8], declaring a 9.4 effective number of bits (ENOB) and a differential non-linearity (DNL), expressed as fraction of least-significant bit (LSB), of ±0.3 LSB. It operates at a sampling frequency f_S=100 kSamples/s, well beyond the Nyquist rate of the signal of interest (the dithering modulation is applied at a frequency $f_{dith} \approx 8$ kHz, therefore each dithering period is sampled 12 times). Since the optical power on the detectors (responsivity 1 A/W) ranges from 1 mW, when the interferometer are not tuned, to less than 100 nW when light is steered away from the monitor PDs, the front-end has to sense dithering signals over a wide input current range. To cope with the limited resolution of the ADC, the amplifying stage features a variable gain, adjusted automatically according to the sampled value. This solution is implemented by modifying the values of the TIA feedback resistor R_F and the GI resistor R_G to increase the amplification R_F/R_G by 4 each time the input current is scaled of the same factor. Supplementary Figure 5b shows the measured transfer function of the TIA for 6 values of R_F, demonstrating a bandwidth around 1 MHz for whatever configuration, enough to detect dithering signals with a frequency around 10 kHz. Montecarlo simulations have been run to assess the repeatability of our design, showing that, for what concerns local loop stability, a phase margin $\phi_M = 75.7°$ ($3\sigma = \pm 5.2°$) is achieved in the most critical configuration, which is $R_F = 5.6\,\mathrm{k\Omega}$. The values sampled by the ADC as a function of the input PD current are instead reported in Supplementary Figure 5c, showing that signals with a dynamic range of more than 50 dB can be correctly acquired by the converter thanks to the adaptive amplification stage. It is also possible to appreciate that, for the highest possible value of amplification, the stage is converting currents up to 1 µA, equivalent to ≈1 µW of optical power, with a resolution of 10 bits. The resulting LSB is thus equivalent to −60 dBm, well below the equivalent optical power generated by the dark current of the PD (between −48 and −38 dBm). Finally, the analog readout chain has been designed targeting, for whatever configuration of the resistive path, an overall root-mean-square (RMS) noise at the output of the gated integrator below 900 µV, that is the amplitude of the ADC quantization error.

The actuator driving circuit comprises a 12-bit digital-to-analog converter (DAC) and a high-current driver. The resolution of the DAC has been chosen to

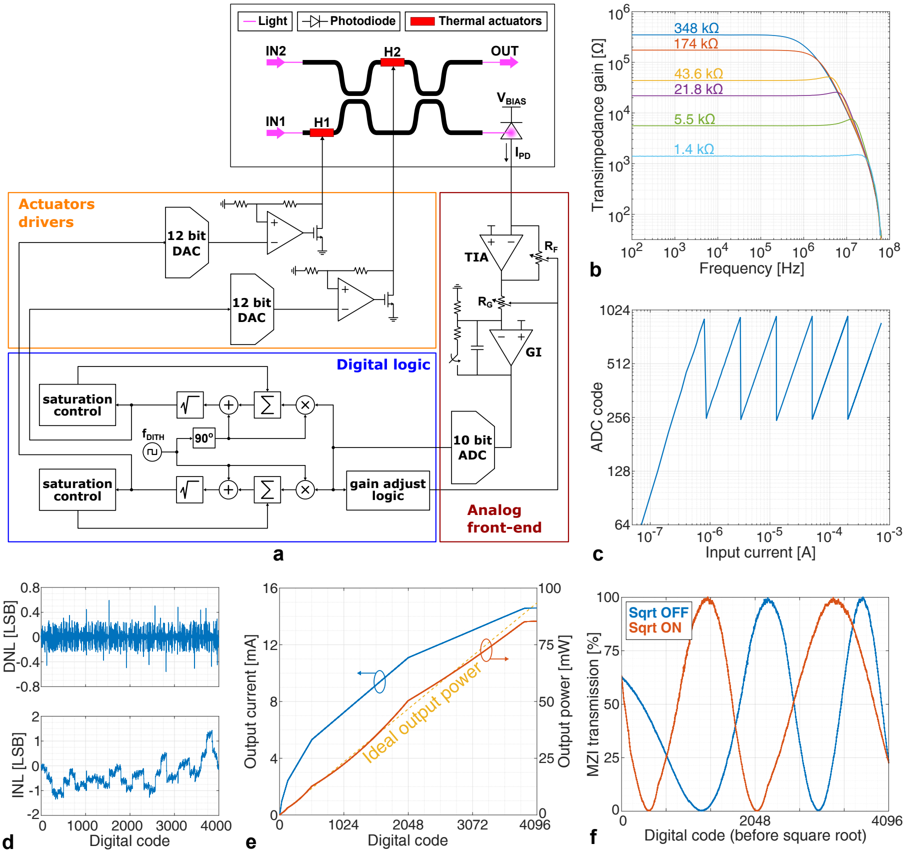

Fig. 5 Electrical characterization of the ASIC. a) Schematic view of the architecture of a single ASIC channel. b) Frequency response of the TIA for different gain values. c) Automatic adjusting of the scale of the ADC over the full range of input current. d) Characterization of the DAC non-linearities and e) validation of the square root approximation. f) MZI transfer function obtained by sweeping the heater voltage, with square root compression on and off.

provide an accuracy of about 1.5 mV on the heaters control voltage. This corresponds to an uncertainty of the phase shift of about 2 mrad in the worst case, which, in an ideal MZI, would lead to negligible crosstalk of about −60 dB. The DAC has been designed with a monotonic transfer function, which guarantees that the sign of the feedback loop is not accidentally inverted during the operation of the ASIC. The DAC characterization in terms of differential and integral non-linearity (INL), expressed as fraction of LSB, is reported in Supplementary Figure 5d, confirming the monotonic behavior of the converter (DNL always below 1 LSB). Since DNL is partially dependent on process and mismatch parameters, a Montecarlo analysis has also been conducted in simulation, concluding that our DAC is affected by an average DNL of 0.09 LSB, with standard deviation $\sigma_{DNL} = 0.33$ LSB, meaning that we can expect more than 99% of the chip samples to exhibit monotonic behavior.

The high-current driver is needed for providing up to 15 mA to the thermal actuators, corresponding to around 4π of phase shift in the MZI transfer function. This wide operating range is required to always ensure convergence of the feedback algorithm, regardless of the initial heater voltage. Being the generated phase shift proportional to the power dissipated by the actuators, a quadratic non-linearity is inherently present in the transfer function from the control voltage to the output optical power. In order to linearize the control loop, an approximated square root digital operation is performed on the digital value fed to the DAC. Such a solution trades precision for compactness, since it occupies only 4% of the area of the digital chip area. Supplementary Figure 5e shows the current provided to the actuators by the driving stage, and the corresponding heater dissipated power, confirming the effective linearization performed by the square root operator. The same result can be observed in Supplementary Figure 5f, which reports the optical power at the output of a MZI as a function of the internal heater voltage, when the square root operation is activated/deactivated. As expected, a more linear phase shift is generated by the chip when the digital compression is activated.

The beneficial effect of the linearization can be predicted by computing the difference in heater dissipated power for a 1 LSB change in the DAC command, at different baseline levels. In our system, a voltage up to $V_H = 6\,\mathrm{V}$ is applied by a DAC with $n = 12$ bits to a heater of nominal resistance $R_H = 400\,\Omega$. Without linearization, a 1 LSB variation around zero results in a power change of $\frac{V_H^2}{2^{2n}}/R_H = 5.4\,\mathrm{nW}$. The same 1 LSB step close to the full-scale range corresponds, in terms of dissipated power, to a variation of $\approx \frac{V_H^2}{2^{n-1}}/R_H = 44\,\mu\mathrm{W}$. This huge difference is the obvious result of the quadratic relation between applied voltage and dissipated power. The consequence is that, without linearization, the number of bits n of the DAC should be chosen accounting for the largest possible step of the heater power. Instead, when the characteristic is linearized, the same resolution across the full V_H swing can be achieved by a DAC with just $n - 1$ bits.

Supplementary section 5: photonic building blocks and PIC loss analysis

The self-aligning beam coupler has been designed in standard Silicon Photonics technology. The chip has been manufactured in an active multi-project wafer run by Advanced Micro Foundry, Singapore [9]. Supplementary Figure 6 summarizes the main photonic building blocks that have been employed in the design of the circuit. The footprint and the main distinctive features of each device are also reported.

The optical antenna array (OOA) has been designed to maximize the received power in presence of turbulence in the free-space link. To do so, the size of the apertures must be comparable to the coherence radius r_0 of the received turbulent beam, they must be separated by at least r_0, and must be as many as possible to minimize geometrical losses [10]. In our case, the OAA has been designed based on simulation results for a scintillation index $\sigma_I^2 = 0.01$ and $r_0 = 4\,\text{cm}$, corresponding to an outdoor link several hundred meters long [11, 12].

DEVICE	LAYOUT	MAIN FEATURES
Waveguide (WG) and thermo-optic phase shifter (PS)	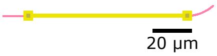 20 μm	WG propagation loss: *~1 db/cm*<br>PS: *TiN heater (80 μm x 2 μm)*<br>PS response time: *~10 μs*
Mach-Zehnder Interferometer (MZI) with 2 phase shifters	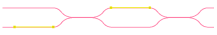 80 μm	Directional coupler (DC) gap: *300 nm*<br>DC length: *40 μm*<br>MZI extintion ratio: *~30 dB*<br>PS efficiency: 25 *mW/π*
Grating coupler (aperture)	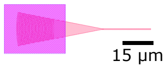 15 μm	Polarization: *transverse-electric (TE)*<br>Beam width: *5° x 9° (HPBW)*<br>Tilt angle: *12°*<br>Aperture size: *29.2 μm x 19.3 μm*
Input optical antenna array	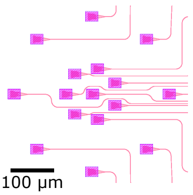 100 μm	Number of apertures: *16*<br>Inner aperture radius: *60 μm*<br>Outer aperture radius: *180 μm*

Fig. 6 Design of the optical beam coupler. Main building blocks used to implement the integrated photonic processors and their relevant characteristics.

The loss breakdown along the entire optical system is computed as follows. Each grating coupler has about 4.5 dB coupling loss when coupled with standard optical fibers, which translates to about 11 dB insertion loss for a fiber-waveguide-fiber coupling (on-chip loss of the photonic processor is around 2 dB). When the free-space beam generated by the fiber collimator is coupled to the 2D array of the photonic processor, a geometrical loss of about 20 dB is

added due to the limited fill factor of the antenna array. This loss can be effectively reduced by improving the fill factor using an array of lenslets at the chip input. To this end, 3D printing techniques, such as two-photon polymerization (TPP), can be used to build custom-designed free-form optical elements directly on top of the photonic chip, thus facilitating optical alignment and reducing assembly and packaging costs [13]. An additional 2 dB loss is due to the aberration of the optical system and possible minor alignment tolerances in the setup. Therefore, the end-to-end loss (from fiber collimator to fiber coupling with output waveguide) is about 30 dB.

Supplementary section 6: ASIC comparison with other electronic controllers

Figure 7 compares the presented ASIC with other electronic controllers for PICs found in the literature. Our chip is much more competitive in terms of area occupation and power dissipation than discrete-components-based solutions [14, 15]. When considering mass production and large markets, the ASIC also results in a lower production cost, including both fabrication and assembly

	[14]	[15]	[18]	[19]	[20]	[21]	**This work**
Photonic device	*Any*	*Any*	*MZI or MRR*	*MRR*	*MRR*	*MRR*	***MZI or MRR***
Sensor type	*Any*	*Any*	*Integrated photodiodes*	*Integrated photodiodes*	*Integrated photodiodes*	*Integrated photodiodes*	***Integrated photodiodes***
Max # of heaters / photonic device	*2*	*2*	*2*	*1*	*1*	*1*	***2***
Technology of the controller	*μC-based discrete components*	*FPGA-based discrete components*	*Silicon Photonics + FPGA*	*45 nm CMOS + FPGA*	*28 nm CMOS*	*28 nm CMOS*	***350 nm CMOS***
Feedback method	-	-	*Digital dithering-based*	*Absolute optical power level search*	*Digital bang-bang*	*Digital bang-bang*	***Digital dithering-based***
Lock point	*Any*	*Any*	*Min/max*	*Any*	*Slope*	*Slope*	***Min/max***
Fully integrated	*No*	*No*	*No*	*No*	*Yes*	*Yes*	***Yes***
Requires calibration	-	-	*No*	*Yes*	*No*	*Yes*	***No***
# of controlled devices	*64*	*16*	*15*	*12*	*1*	*4*	***8***
Power / photonic device	*≈ 0.5 W*	*≈ 0.5 W*	*≈ 0.1 W*	*≈ 0.1 W*	*16 mW*	-	***10 mW***
Response time	-	*12 ms*	*10 ms*	*100 ms*	*0.5 ms* *	-	***10 ms***
Size	*> cm^2*	*> cm^2*	*0.25 x 1.2 mm^2* **	*0.22 x 2.28 mm^2* **	*1.5 x 1.5 mm^2* ***	*0.57 mm^2*	***3 x 4.1 mm^2***
Demonstrated application	*Reconfigurable optical processor*	*Reconfigurable optical processor*	*Reconfigurable optical processor*	*Single photon generation*	*Optical transmitter*	*Optical transmitter*	***Free-space optical receiver***

* simulation ** without FPGA *** including TX circuits

Fig. 7 Comparison with the state-of-the-art. The table summarizes the main features of electronic controllers found in the literature. The presented chip provides compact area, low power consumption, fast response time and calibration-free control law, thus satisfying all the general requirements of electronic controllers.

of the complete electronic-photonic system. However, since a specific control feedback law is targeted, this comes at the price of lower flexibility and adaptability to different applications and photonic chips. Our chip is suited only to programmable PICs whose functionality can be defined by completely switching on/off local optical paths. Even though this includes many possible applications (e.g. universal beam couplers and coherent adders [10], optical routers [16], wavelength-selective filters and multiplexers [17]), a different control approach might be needed in other cases, requiring an update of the digital architecture of the ASIC. A new chip could be conceived to target both minimization/maximization and partial splitting of light, thus generalizing the control paradigm to more photonic architectures.

A possibility for maintaining a good level of flexibility while reducing area and cost is to integrate only part of the control circuitry, while still relying on external digital logic [18, 19]. This approach allows simple prototyping and optimization of the control strategy without the need for bulky electronics, especially if the readout and driving circuits (TIAs, ADCs and DACs) are integrated and only an external digital processor is required. However, when the feedback law is well-defined or when an ASIC is already needed to operate a PIC, a fully-integrated solution is more effective. Previous demonstrations of this kind [20, 21] focused only on MRRs and optical transceivers. Our chip extends the control approach to the case of MZIs, which is more complex since two independent actuators need to be properly driven. Therefore, it enables a broader range of applications.

It is worth noticing that the presented ASIC features a response time similar to other implementations, meaning that the integration of front-end and driving circuits does not impair the control performance. In addition, the calibration-free feedback loop does not require time-consuming sample-specific preliminary measurements and it is not affected by the working and environmental conditions of the PIC. This is a relevant advantage of the proposed circuit over other controllers found in the literature. Finally, thanks to its digital I/O interface, the current ASIC could also be used in combination with external digital processors, even though it was not optimized for this purpose. Indeed, the ADCs output stream can be read and elaborated by an external controller and fed back to the on-chip DACs to close a hybrid control loop. However, due to the limited read and write speed of the internal registers, as well as the latency of the communication, this mode of operation is suitable only for those applications where a relatively slow response time of the feedback loop can be accepted.

Supplementary references

[1] Zanetto, F., Grimaldi, V., Toso, F., Guglielmi, E., Milanizadeh, M., Aguiar, D., Moralis-Pegios, M., Pitris, S., Alexoudi, T., Morichetti, F., Melloni, A., Ferrari, G., Sampietro, M.: Dithering-based real-time control of cascaded silicon photonic devices by means of non-invasive detectors. IET Optoelectronics **15**(2), 111–120 (2021)

[2] Padmaraju, K., Logan, D.F., Shiraishi, T., Ackert, J.J., Knights, A.P., Bergman, K.: Wavelength locking and thermally stabilizing microring resonators using dithering signals. Journal of Lightwave Technology **32**(3), 505–512 (2013)

[3] Miller, D.A.B.: Self-aligning universal beam coupler. Optics express **21**(5), 6360–6370 (2013)

[4] Gazman, A., Manzhosov, E., Browning, C., Bahadori, M., London, Y., Barry, L., Bergman, K.: Tapless and topology agnostic calibration solution for silicon photonic switches. Optics express **26**(25), 32662–32674 (2018)

[5] Pérez-López, D., López, A., DasMahapatra, P., Capmany, J.: Multipurpose self-configuration of programmable photonic circuits. Nature communications **11**(1), 6359 (2020)

[6] Catalá-Lahoz, C., Pérez-López, D., Huy-Ho, T., Capmany, J.: Self-configuring programmable silicon photonic filter for integrated microwave photonic processors. APL Photonics **8**(11) (2023)

[7] Bandyopadhyay, S., Hamerly, R., Englund, D.: Hardware error correction for programmable photonics. Optica **8**(10), 1247–1255 (2021)

[8] ams-OSRAM AG. https://ams-osram.com/

[9] Advanced Micro Foundry Pte Ltd. https://www.advmf.com/

[10] Martinez, A.I., Cavicchioli, G., Seyedinnavadeh, S., Zanetto, F., Sampietro, M., D'Acierno, A., Morichetti, F., Melloni, A.: Self-adaptive integrated photonic receiver for turbulence compensation in free space optical links. Scientific Reports **14**(1), 20178 (2024)

[11] Zhu, X., Kahn, J.M.: Free-space optical communication through atmospheric turbulence channels. IEEE Transactions on communications **50**(8), 1293–1300 (2002)

[12] Cox, M.A., Mphuthi, N., Nape, I., Mashaba, N., Cheng, L., Forbes, A.: Structured light in turbulence. IEEE Journal of Selected Topics in Quantum Electronics **27**(2), 1–21 (2020)

[13] Yu, S., Ranno, L., Du, Q., Serna, S., McDonough, C., Fahrenkopf, N., Gu, T., Hu, J.: Free-form micro-optics enabling ultra-broadband low-loss off-chip coupling. Laser & Photonics Reviews **17**(6), 2200025 (2023)

[14] Shawon, M.J., Saxena, V.: A silicon photonic reconfigurable optical analog processor (SiROAP) with a 4x4 optical mesh. In: 2023 IEEE International Solid-State Circuits Conference (ISSCC), pp. 222–224 (2023)

[15] Guglielmi, E., Carminati, M., Zanetto, F., Annoni, A., Morichetti, F., Melloni, A., Sampietro, M., Ferrari, G.: 16-channel modular platform for automatic control and reconfiguration of complex photonic circuits. In: 2017 IEEE International Symposium on Circuits and Systems (ISCAS), pp. 1–4 (2017)

[16] Ji, R., Yang, L., Zhang, L., Tian, Y., Ding, J., Chen, H., Lu, Y., Zhou, P., Zhu, W.: Microring-resonator-based four-port optical router for photonic networks-on-chip. Optics express **19**(20), 18945–18955 (2011)

[17] Dong, P.: Silicon photonic integrated circuits for wavelength-division multiplexing applications. IEEE Journal of Selected Topics in Quantum Electronics **22**(6), 370–378 (2016)

[18] Zanetto, F., Toso, F., Grimaldi, V., Petrini, M., Martinez, A., Milanizadeh, M., Perino, A., Morichetti, F., Melloni, A., Sampietro, M.: Time-multiplexed control of programmable silicon photonic circuits enabled by monolithic CMOS electronics. Laser & Photonics Reviews **17**(11), 2300124 (2023)

[19] Kramnik, D., Wang, I., Ramesh, A., Fargas Cabanillas, J.M., Gluhović, D., Buchbinder, S., Zarkos, P., Adamopoulos, C., Kumar, P., Stojanović, V.M., Popović, M.A.: Scalable feedback stabilization of quantum light sources on a CMOS chip. arXiv preprint arXiv:2411.05921 (2024)

[20] Li, H., Balamurugan, G., Kim, T., Sakib, M.N., Kumar, R., Rong, H., Jaussi, J., Casper, B.: A 3D-integrated silicon photonic microring-based 112-Gb/s PAM-4 transmitter with nonlinear equalization and thermal control. IEEE Journal of Solid-State Circuits **56**(1), 19–29 (2021)

[21] Sharma, J., Xuan, Z., Li, H., Kim, T., Kumar, R., Sakib, M.N., Hsu, C.-M., Ma, C., Rong, H., Balamurugan, G., Jaussi, J.: Silicon photonic microring-based 4 x 112 Gb/s WDM transmitter with photocurrent-based thermal control in 28-nm CMOS. IEEE Journal of Solid-State Circuits **57**(4), 1187–1198 (2022)
